# Nanosecond-Lived Excimer Observation in a Crystal of a Rhodium(I) Complex via Time-Resolved X-ray Laue Diffraction

**DOI:** 10.1021/acs.jpclett.4c02476

**Published:** 2024-10-09

**Authors:** Piotr Łaski, Lerato Bosman, Jakub Drapała, Radosław Kamiński, Dariusz Szarejko, Patryk Borowski, Andreas Roodt, Robert Henning, Alice Brink, Katarzyna N. Jarzembska

**Affiliations:** † Department of Chemistry, University of Warsaw, Żwirki i Wigury 101, 02-089 Warsaw, Poland; ‡ Department of Chemistry, University of the Free State, Nelson Mandela Drive, Bloemfontein 9301, South Africa; § Faculty of Chemistry, Warsaw University of Technology, Noakowskiego 3, 00-664 Warsaw, Poland; ∥ Center for Advanced Radiation Sources, University of Chicago, 5734 South Ellis Avenue, Chicago, Illinois 60637, United States

## Abstract

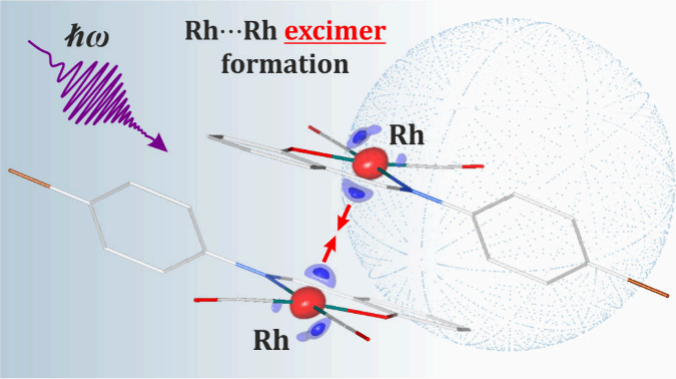

The rare observation of transient Rh···Rh excimer formation in a single crystal is reported. The estimated excited-state lifetime at 100 K is 2 ns, which makes it the shortest-lived small-molecule species caught experimentally using the laser-pump/X-ray-probe time-resolved Laue method. Upon excitation with 390 nm laser light, the intermolecular Rh···Rh distance decreases from 3.379(4) to 3.19(1) Å, and the metal–metal contact gains more bonding character. On the basis of the experimental results and theoretical modeling, the structural changes determined with 100 ps time resolution reflect principally the S_0_ → S_1_ electronic transition.

Profound investigations of light–matter interactions are indispensable for understanding the mechanisms of crucial (bio)chemical processes, the nature of excited states, and structural dynamics. Such knowledge can be successfully applied to the design of novel effective functional materials for applications in optoelectronics, solar energy conversion systems, storage devices, sensors, etc.^1−5^ Because many such materials are solid-state materials, conducting studies using their applicable form or at least as a simplified model would be desirable. In this respect, crystals constitute convenient model systems, as they can be relatively easily studied using crystallographic methods. Nevertheless, to trace short-lived transient species, advanced approaches have to be applied, such as laser-pump/X-ray-probe methods combined with serial crystallography,^6−8^ or the “pink”-beam Laue technique.^9−14^ To achieve the required fine time resolution, such experiments are performed at synchrotron or XFEL sources, where ultrashort (approximately femtoseconds to picoseconds) X-ray pulses can be generated. A number of studies of this kind have already been conducted for macromolecular samples;^13,15−20^ however, due to the development of data analysis tools, some small-molecule crystals have also been quite successfully examined to date.^9,10,12,21−34^

In this work, we have focused our attention on a newly synthesized rhodium(I)-based potential precatalyst (hereafter **Rh-4-Br**) for model Monsanto reactions.^35−38^ The rhodium Monsanto process has played a significant role in the homogeneous catalytic reaction involving the carbonylation of methanol to acetic acid with an annual global use of several million tons of acetic acid.^39−41^

The distorted-square-planar molecular structure of **Rh-4-Br** is shown in Figure 1a. The rhodium atom is coordinated by two carbonyl groups and by a monoanionic N,O-donor bidentate ligand. The phenyl ring connected to the N1 atom is substituted with a bromine atom in the *para* position. The compound crystallizes in space group *P*2_1_/*n*, with one molecule in the asymmetric unit. The strongest interacting dimeric motif in the solid state is illustrated in the crystal structure, characterized by an interaction energy of approximately −74 kJ mol^–1^ (Table S3.2), as shown in Figure 1b. It consists of two center-of-symmetry-related molecules and is stabilized mainly by the d^8^–d^8^ (d_*z*^2^_-type) Rh···Rh metallophilic contact [metal–metal distances of 3.379(4) Å] and two C15–H15···O3 hydrogen-bond-type interactions between the bromine-substituted phenyl rings and the oxygen atom from the Rh coordination sphere of the adjacent molecule. Importantly, these dimers constitute discrete motifs in the crystal structure (Figure S2.1a), as the Rh···Rh interactions do not propagate further in space, a rare occurrence for many complexes of this type.^42,43^ The other side of the metal center is surrounded by two bromine atoms of the two molecules located above (Supporting Information).

**Figure 1:**
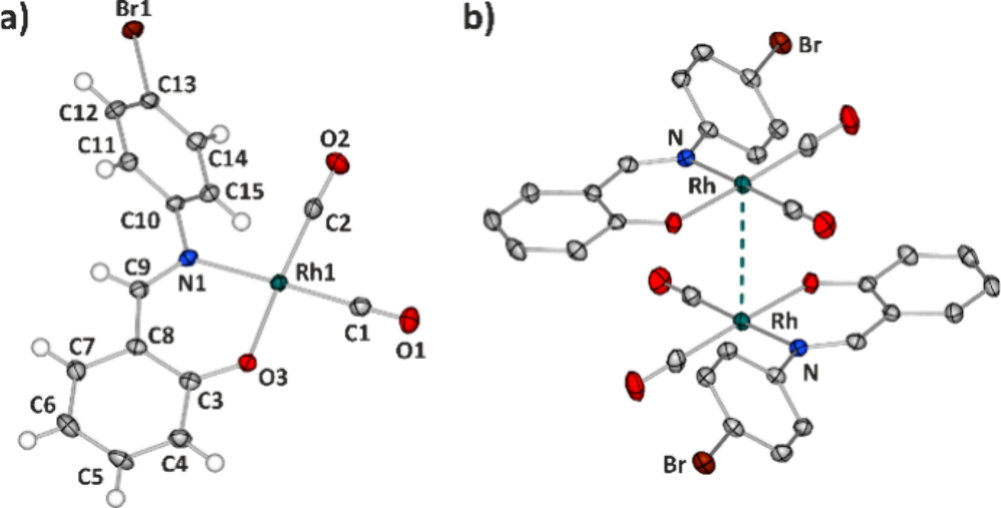
(a) Molecular structure of **Rh-4-Br**. (b) Main dimeric motif in the crystal structure of **Rh-4-Br** [note the Rh···Rh distance is 3.379(4) Å]. Thermal motion is shown as ellipsoids at the 50% probability level.

The shortest intermolecular Rh···Br distances amount to 4.206(5) and 4.348(5) Å, and the interaction energies of the respective dimers, stabilized also by hydrogen-bond-type interactions, are equal to −26.3 and −40.9 kJ mol^–1^, respectively. With respect to the crystal architecture, slightly undulated molecular layers parallel to the (103) crystal plane can be distinguished (Figure S2.1b).

Platinum-group transition-metal coordination compounds often exhibit interesting spectroscopic behavior, which can be significantly affected by the metallophilic interactions if formed.^44−48^ The luminescence shown by **Rh-4-Br** can also, to some extent, be associated with the d_*z*^2^_-type Rh···Rh contacts. Fluorescence is rather weak in the solid state, with the maximum at ∼560 nm (Table 1). The estimated lifetime of the emissive state is <1 ns at room temperature (∼700 ps) and increases with a decrease in temperature reaching ∼2 ns at 100 K. No thermally activated delayed fluorescence (TADF)^49^ was detected (Supporting Information).

**Table 1:** Solid-State Emission Maxima and Lifetimes for **Rh-4-Br**

temperature, *T* (K)	emission maximum, *λ*_em_^max^ (nm)	emission lifetime, *τ* (ns)
room temperature	566	0.70(1)
250	561	0.77(1)
200	558	1.25(1)
150	558	1.70(1)
100	557	2.01(1)

The theoretical computations were performed by using the density functional theory (DFT) method (Supporting Information). The used range-separated CAM-B3LYP functional^50^ should take into account possible charge transfer occurring upon excitation, which is important once a dimeric motif is considered. According to the time-dependent DFT-derived vertical electronic transitions, the lowest singlet–singlet transition occurs at ∼360 nm. It involves a number of molecular orbitals with the most significant contributions from the HOMO–2 → LUMO and HOMO–2 → LUMO+2 transitions, and noteworthy HOMO → LUMO and HOMO → LUMO+2 components (Figure S3.1). The HOMO–2 orbital is located principally on the Rh centers (mainly d_*z*^2^_ atomic orbitals) and has an antibonding character. HOMO also covers the Rh atomic orbitals and has a similar antibonding nature; however, it is additionally visibly spread over the heterocyclic ligand fragment. In turn, the LUMO orbital involves the heterocyclic fragment of the ligand, and to a lesser extent the Rh atomic orbitals (mainly d_*xy*_), whereas LUMO+2 shows more emphasized metal–metal bond character (Figure S3.1f). Overall, the S_0_ → S_1_ electronic transition is a mixture of π → π* excitation and metal-to-ligand charge transfer (MLCT) with a metal-to-metal bond CT contribution. Relatively close in energy, at ∼345 nm, a similar in nature but brighter S_0_ → S_3_ transition can be found (Table S3.1). In this case, the MLCT character is much more dominant. Also, the lowest-energy singlet–triplet transition (S_0_ → T_1_, ∼476 nm) resembles the S_0_ → S_3_ transition in character, though it lacks contributions from HOMO–2 and LUMO+2 involving the Rh···Rh region most. The calculated ultraviolet–visible spectrum well reflects features of the respective experimental solid-state data (Figure S4.1). The latter is more spread out and shifted toward lower energies, which is typical for solid-state absorption spectra.

In light of the information presented above, we expect some structural changes in the central region of the studied molecule once the system is excited with the 390 nm laser light matching the experimental solid-state band with respect to the S_0_ → S_1_ electronic transition. Thus, the time-resolved (TR) laser-pump/X-ray-probe Laue diffraction experiment was carried out at the BioCARS beamline in APS, allowing for ∼100 ps single-X-ray-pulse diffraction.^20,51^ The X-ray diffraction signals were collected both after (“ON”, pump–probe delay set to 100 ps; laser-pulse duration of 38 ps) and without (“OFF”) laser exposure, while further data treatment was based on the intensity ratios (*R*_ON/OFF_ = *I*_ON_/*I*_OFF_).^52^ The collected data were integrated using our GPU-accelerated one-dimensional seed-skewness algorithm (Supporting Information).^53^ Further processing^24,54−59^ and merging of four best-quality data sets yielded a single data set of ∼50% overall completeness. Due to the relatively small excimer population, charge density changes can be reliably assessed only fairly close to the heavy atoms (here Rh and Br atoms), while the statistical noise and Fourier-rippling effects for less complete data sets overshadow the possible signal in the remaining part of the molecule (for details, see the Supporting Information). The resulting photodifference map^60,61^ (*F*_ON_^100 ps^ – *F*_OFF_) illustrating the observed electron density changes upon laser light excitation is presented in Figure 2.

**Figure 2:**
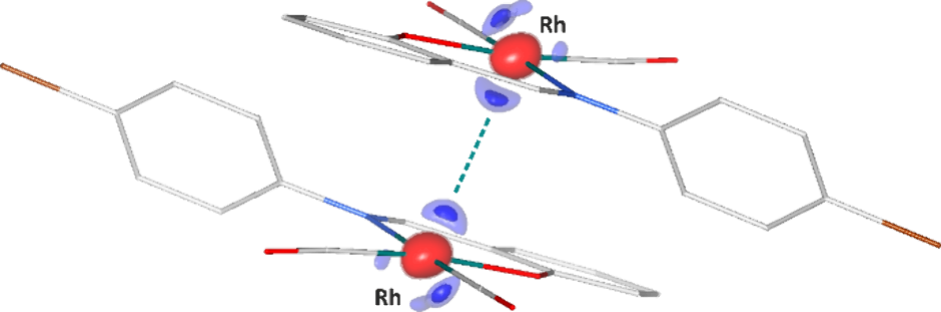
Photodifference map (*F*_ON_^100 ps^ – *F*_OFF_) of **Rh-4-Br** showing atomic shifts in the S_1_ excited state superimposed onto the S_0_ ground-state geometry. Solid isosurfaces, ±0.50 e Å^–3^; semitransparent, ±0.41 e Å^–3^; blue for positive and red for negative.

The significant accumulation of electrons in the region between the two Rh centers and typical depletion of electron density at atomic positions suggest temporary contraction of the metal–metal bond after excitation and increased vibration of all atoms due to the laser-induced increase in temperature. A weaker signal on the other side of the metal center in the direction of the neighboring Br atom can be related to a shift of both the adjacent molecules toward each other upon excitation (Figure S5.3). Indeed, it is also accompanied by accumulation of some electron density at the respective side of the Br atom. Pairs of closest bromine atoms also seem to move slightly toward each other upon excitation. It should also be noted that on the basis of the photo-Wilson plot analysis,^62−64^ the increase in temperature upon excitation was estimated to be ∼4 K (Figure S5.6).

To determine the experimental geometry of the short-lived excited-state species and verify the presumptions described above, a response ratio [η = (*I*_ON_ – *I*_OFF_)/*I*_OFF_ = *R*_ON/OFF_ – 1] structure refinement was conducted.^65−67^ Given the moderate data completeness, it was crucial to first estimate the population of the excited state so it can be set at a fixed value during further refinement steps. An excited-state population of 1% assures the lowest discrepancy ratio-based *R*_*R*_ factor^68^ (Figure S5.4), whereas the most reasonable results were obtained when only the Rh atoms were refined freely. Such an approach is sensible, taking into account the very small excited-state population and data completeness. As a result, a notable signal is obtained only for electron-rich heavy atoms such as Rh or Br. Indeed, apart from the electron density peaks in the vicinity of the Rh centers, some significant electron density redistribution is also visible for the bromine atoms. However, the refinement of Br strongly affects the position of the organic part of the molecule that cannot be refined reliably (Supporting Information). In all of the tested structural models, the Rh···Rh contact shortens significantly. The intermolecular Rh···Rh distance decreases from 3.379(4) to 3.19(1) Å (i.e., by ∼6%) in the most trustworthy model, leading to metallophilic interaction strengthening and formation of the excimer species. The character of this transition, spectroscopic features, and structural changes are in agreement with the time-dependent DFT results for the S_0_ → S_1_ transition and the QM/MM modeling (Figure S3.3).^69^ Its nature is illustrated well by the transition density map and atomic charges (Supporting Information). Nonetheless, the minor contribution from the T_1_ excited state cannot be completely excluded. The comparison of the QM/MM and isolated-molecule calculation results with experiments shown in Table 2 indicates that the Rh···Rh distance should decrease upon excitation of excited singlet state S_1_ and even more for a potential triplet state T_1_. In the case of the optimized isolated dimer, its components are slightly farther apart in the ground state, while upon excitation to S_1_ or T_1_, the Rh···Rh distance shortens significantly more compared to the solid-state results, by ∼0.4 Å for S_1_ and >0.55 Å for T_1_. It should also be noted that although the QM/MM-optimized structure for the ground state does not fully match the experimental value, the chosen level of theory yields a sensible excited-state geometry (Supporting Information). Indeed, the experimental and predicted Rh···Rh distances in the S_1_ state are statistically consistent. The investigations show that reliable experimental results are extremely important in the case of modeling of excited-state species (Supporting Information).

**Table 2:** Comparison of Rh···Rh Distances in the Ground (S_0_) and Excited (S_1_ and T_1_) States^a^

	Rh···Rh distance, *d*_Rh···Rh_ (Å)		
electronic state	experimental	QM/MM	isolated dimer
S_0_	3.379(4)	3.486	3.495
S_1_	3.19(1)	3.205	2.971
T_1_	–	3.195	2.816

a Theoretical geometries of DFT(CAM-B3LYP) with 6-31G** (C, H, O, N, Br)/LANL2DZ (Rh); crystal environment modeled with the Universal Force Field approximation in QM/MM.

Overall, the experimental results indicate the formation of an excimer upon near-ultraviolet light irradiation of the **Rh-4-Br** crystal. To date, only one other excimer was detected using the (monochromatic) TR diffraction technique.^70^ However, in that case, the bonding situation is far more complex due to the presence of infinite molecular stacks in the crystal structure; thus, the nature of the laser-generated species remains unclear. The excited-state population of **Rh-4-Br** was estimated to be ∼1%, while its lifetime was estimated to be 2 ns at 100 K (no TADF signal was detected). Hence, this is the shortest-living species caught in a time-resolved X-ray Laue experiment so far.^21−25,27,28,31−33,71^ This was further evidenced during the pump–probe Laue experiment by the lack of a detectable differential signal 1 ns after laser excitation. Theoretical computations indicated the presence of mixed MLCT and π → π* transitions and some metal-to-metal bond CT contribution. Nevertheless, as opposed to other works on the Rh···Rh distance contractions,^24−26^ on the basis of the spectroscopic features, short TR Laue signal, and theoretical predictions, the refined excited-state model of **Rh-4-Br** can most likely be attributed to the lowest-lying S_1_ singlet state. In the previously reported cases of Rh···Rh shortening, often the information about the systems was not fully consistent (e.g., the ES lifetime was determined at a temperature different from that of the TR Laue experiment), and the S_1_ excited state was not taken into consideration, even though the 100 ps time delay with the 100 ps-long probe was applied. In this work, excited states S_1_ and T_1_ were modeled and analyzed, and the increase in the temperature of the sample upon excitation was estimated. The study shows that using the TR Laue method and newly developed processing schemes, it is possible to detect and refine very short-lived excited-state species with close-to-residual populations. We believe that with the ongoing emergence of XFELs, our efforts will contribute to the development of the methods and the design of approximately femtosecond pump–probe experiments in the future.

## Supporting Information Available

Structural, photocrystallographic, spectroscopic, and computational details and relevant figures (PDF)
Crystallographic information (CIF)

## Supplementary Material

jz4c02476_si_001.pdf

jz4c02476_si_002.cif

## Abbreviations

XFEL: X-ray free-electron laser
HOMO: highest occupied molecular orbital
LUMO: lowest unoccupied molecular orbital
CT: charge transfer
APS: Advanced Photon Source
GPU: graphical processing unit
QM/MM: quantum mechanics/molecular mechanics
