# Supplementary material for: Nanosecond-Lived Excimer Observation in a Crystal of a Rhodium(I) Complex via Time-Resolved X-ray Laue Diffraction
Source: J Phys Chem Lett. 2024 Oct 9;15(41):10301–6. doi: 10.1021/acs.jpclett.4c02476 (PMC11492376; doi:10.1021/acs.jpclett.4c02476)
Supplement: Supplementary file 1 — jz4c02476_si_001.pdf [file jz4c02476_si_001.pdf]

SUPPORTING INFORMATION

Nanosecond-Lived Excimer Observation in a Crystal of a Rhodium(I) Complex via Time-Resolved X-ray Laue Diffraction

Piotr Łaski,[†] Lerato Bosman,[‡] Jakub Drapała,[†,₴] Radosław Kamiński,[†]
Dariusz Szarejko,[†] Patryk Borowski,[†] Andreas Roodt,[‡] Robert Henning,[¥]
Alice Brink,[*,‡] Katarzyna N. Jarzembska [*,†]

[†] Department of Chemistry, University of Warsaw, Warsaw, Poland

[‡] Department of Chemistry, University of the Free State, Bloemfontein, South Africa

[₴] Faculty of Chemistry, Warsaw University of Technology, Warsaw, Poland

[¥] Center for Advanced Radiation Sources, University of Chicago, Chicago, Illinois, United States

* Corresponding authors: Alice Brink (brinka@ufs.ac.za)
Katarzyna N. Jarzembska (katarzyna.jarzembska@uw.edu.pl)

Table of Contents

1. Synthesis S2
2. X-ray crystallography S2
  2.1. In-house data collection S2
  2.2. CSD search S4
3. Theoretical calculations and modelling S5
4. Solid-state spectroscopy S12
5. Time-resolved X-ray Laue diffraction S15
  5.1. Data collection S15
  5.2. Data processing S16
  5.3. Photodifference maps S16
  5.4. Refinement S17
6. References S25

1. Synthesis

$RhCl_3 \cdot 3H_2O$ (0.01003 g, 0.0378 mmol) was refluxed in 2 mL of DMF until the red color was observed to turn yellow (approx. 30 min.). The rhodium dimer (2 mL) was added to 4-bromosalicylideneaniline (0.0136 g, 0.0492 mmol) and stirred for 5 min., the product was precipitated by addition of ice water. The solid was filtered, and the precipitate recrystallized using dichloromethane. Yellow crystals were obtained (0.0129 g, 78.6%). FTIR (ATR) in cm^{-1}: 2066 (ν CO) 1990(ν CO). ^{1}H NMR (400.13 MHz, $CDCl_3$) δ (ppm) 12.94 (1H, s, C=NH), 7.49–6.69 (8H, m, Ar–H); ^{13}C NMR (100.61 MHz, $CDCl_3$) δ (ppm) 164.8, 162.1, 149.7, 133.14, 129.15, 129.3, 128.5, 127.9, 128.3, 128.2, 118.8–119.20 (2C, s), 116.9.

2. X-ray crystallography

2.1. In-house data collection. The ambient pressure reflection data were collected on a Bruker AXS D8 Quest diffractometer, equipped with a graphite monochromator using a Mo K_α X-ray generator (λ = 0.71073 Å). Data was collected utilizing both ϕ and ω scans at a temperature of 100 K. *COSMO* procedure (Bruker AXS, 2003) was utilized for multiple hemisphere data collection of the reciprocal space. Bruker AXS *SAINT-PLUS*, *XPREP* and *SADABS* (Bruker AXS, 2004, 2016) programs were employed for frame integration and further data reduction. *SHELXT* (Sheldrick, 2015) was used to solve the crystal structure through intrinsic phasing method. *OLEX2* (Dolomanov *et al.*, 2009) and *SHELXL* (Sheldrick, 2008) were used for the refinement of the crystal structure. The hydrogen atoms were positioned geometrically and refined using a riding model: C–H distances were set to 0.95 Å, and the H-atom isotropic displacement parameters were fixed at $U_{\mathrm{iso}}^{\mathrm{H}} = 1.2 \cdot U_{\mathrm{eq}}^{\mathrm{C}}$.

Table S2.1. Selected X-ray data collection, processing and refinement parameters for the studied **Rh-4-Br** compound.

Moiety formula	$C_{15}H_9BrNO_3Rh$	V / Å^3	1415.1(2)
Moiety formula mass, M_r / a.u.	434.05	d_{calc} / g·cm^{-3}	2.037
Crystal system	monoclinic	θ range	2.63–28.34°
Space group	$P2_1/n$ (No. 14)	Absorption coefficient, μ / mm^{-1}	4.038
Z	4	No. of reflections collected / unique	65357 / 3517
F_{000}	840	R_{mrg}	5.43%
Crystal color & shape	yellow plate	No. of reflections with $I > 2\sigma(I)$	3309
Crystal size / mm^3	0.05 × 0.11 × 0.11	No. of parameters	190
T / K	100	$R[F]$ $(I > 2\sigma(I))$	2.09%
a / Å	8.8807(9)	$R[F^2]$ (all data)	5.06%
b / Å	15.1873(15)	$\varrho_{res}^{min/max}$ / e Å^{-3}	−0.64 / +0.57
c / Å	10.9202(11)	CCDC code	2101084
β / °	106.098(4)		

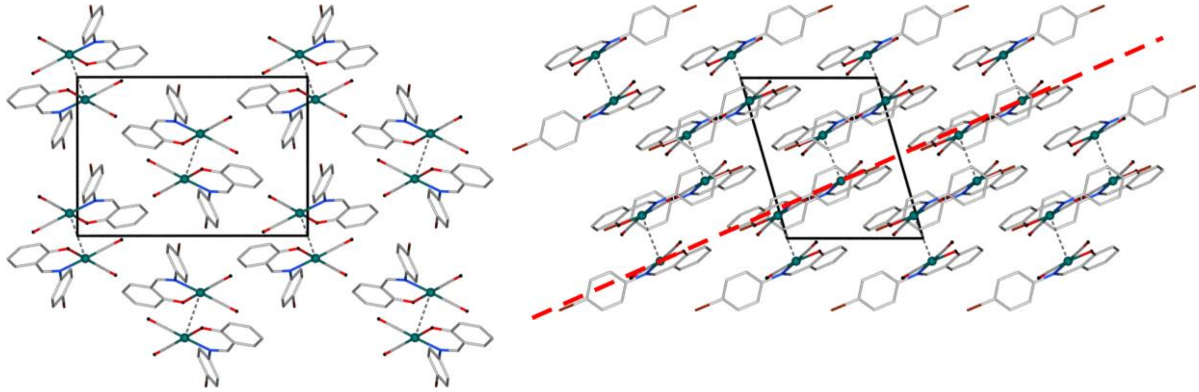

(*a*) (*b*)

Figure S2.1. (a) Packing of molecules in the crystal structure of **Rh-4-Br** showing dimeric motifs (view along X axis). Rh⋯Rh interactions arrange along either $[12\bar{8}]$ or $[1\bar{2}\bar{8}]$ crystallographic directions. (b) Packing of **Rh-4-Br** molecules shown along Y axis. Layered structure is visible; red dashed line represents $(\bar{1}03)$ crystal plane.

2.2. CSD search. Short summary of Rh(*O,O'*)-type crystal structures available in the Cambridge Structural Database (ver. 5.45 update) (Allen, 2002, Groom *et al.*, 2016). Search is specific to Rh(*O,O'*)-β-diketone with pseudo 5-cyclic coordination to the metal center. Three type of metal-metal chains can be distinguished:

a. Structures with continuous infinite chain formed through Rh···Rh interactions. REFCODES: ACABRH02 (Huq & Skapski, 1974), ACABRH03 (Varshavsky *et al.*, 2018), BTFARH (Leipoldt *et al.*, 1977), BUPCUK (Schurig *et al.*, 1983), DEXHUJ (Schurig *et al.*, 1985), EDOYAZ (Pretorius & Roodt, 2012), QASWOZ and QASWUF (Conradie *et al.*, 2017), TIRZOL (Conradie & Conradie, 2013).

b. Dimers constitute discrete motifs which appear to propagate in an offset fashion into space to form steps by alternating Rh···Rh interactions followed by C–H···π; π···π or similar interactions between the ligand moieties. REFCODES: EPUGEC (Stuurman *et al.*, 2011), KAKYUR (Conradie *et al.*, 2005) – caused by ferrocene moiety, KEFJOV (Mochida *et al.*, 2006) – caused by hydroxyphenalenone moiety, VAVJUX (Schurig *et al.*, 1989) – caused by hexafluoroglutarylbis-(1R)-camphorate moiety, WULSUT (Pretorius *et al.*, 2014) – the chains are offset in steps and may fall into an intermediate category between **b** and **c**.

c. Dimers constitute discrete motifs not propagating further in space. This group includes our new structure **Rh-4-Br**. REFCODES: OJEWIK (Hopmann *et al.*, 2010), PUPDIP (Storch *et al.*, 2015).

3. Theoretical calculations and modelling

Calculations for the purpose of the current study were performed with the DFT method using the CAM-B3LYP functional and mixed 6-31G** (C, H, O, N, Br atoms) and LANL2DZ (Rh atom) basis set level of theory (hereafter abbreviated as 6-31G**-LANL2DZ), using the *GAUSSIAN* package (ver. 16) (Frisch *et al.*, 2016) in order to derive the theoretical UV-Vis spectrum of **Rh-4-Br** and molecular orbitals contributing to the lowest-energy electronic transitions, *i.e.* $S_0 \rightarrow S_1$ (λ = 358.37 nm, f = 0. 0959) and $S_0 \rightarrow T_1$ (λ = 475.99 nm). Calculations were performed for the best-stabilized molecular dimer in the crystal structure exhibiting metallophilic interactions. While energetically close, the next two singlet-singlet transitions (*i.e.* $S_0 \rightarrow S_2$ and $S_0 \rightarrow S_3$) should not be significantly involved in the excimer formation. The $S_0 \rightarrow S_2$ transition is almost dark, with the oscillator strength lower than 1×10^{-4}, whereas the $S_0 \rightarrow S_3$ transition involves molecular orbitals which are less likely to be responsible for the experimentally observed Rh···Rh distance shortening. The molecular orbitals most significantly involved in the $S_0 \rightarrow S_1$ singlet transition (HOMO−2 → LUMO+2, HOMO−2 → LUMO) and the triplet transition (HOMO → LUMO, HOMO−1 → LUMO+1) are described in Table S3.1 and depicted graphically in Figure S3.1. Both of these transitions exhibit MLCT character with some notable contribution of the $\pi \rightarrow \pi^*$ type transitions on ligands. The $S_0 \rightarrow S_1$ transition density map is shown in Figure S3.2.

Molecular geometries in the S_0, S_1, and T_1 electronic states were optimized as isolated dimers at the DFT(CAM-B3LYP)/6-31G**-LANL2DZ level of theory. These optimizations have resulted in geometries with the Rh···Rh distances of 2.971 Å for the S_1 state 2.816 Å for the T_1 state, respectively. However, for the case of isolated molecules, the predicted shortening of Rh···Rh distance can be overexpressed, and as such, in order to account for crystal interactions, a model of the molecular environment in a crystal was considered by using the quantum-mechanics / molecular-mechanics (QM/MM) approach (Kamiński *et al.*, 2010, Vreven *et al.*, 2006). In the QM/MM approach, the central moiety (the Rh···Rh dimer) was treated at the DFT(CAM-B3LYP)/6-31G**-LANL2DZ level of theory, while the molecular shell was approximated with the Universal Force Field (UFF) (Rappé *et al.*, 1992), using the Hirshfeld charges (Hirshfeld, 1977) obtained at the same level of theory for the **Rh-4-Br** monomer. The molecular shell, consisting of a cluster of molecules in a 15 Å radius around the central species, was constructed using *CLUSTERGEN* software (Kamiński *et al.*, 2013), with X−H bond lengths (X = non-hydrogen atom) set to neutron-

normalized values (Allen & Bruno, 2010, Allen *et al.*, 1987). The molecular cluster is presented in Figure S3.3. The geometry optimization process overview with regard to the Rh⋯Rh distance at each optimization step is presented in Figures S3.4. The Rh⋯Rh distances of 3.205 Å (difference between excited and ground states: Δ_{ES-GS} = −0.281 Å) and 3.195 Å (Δ_{ES-GS} = −0.291 Å) were predicted for S_1 and T_1 states, respectively.

For interaction energy calculations for selected molecular motifs, the counterpoise method implemented in *GAUSSIAN* (ver. 16) was applied. The interaction energy was calculated at the DFT(CAM-B3LYP)/6-31G**-LANL2DZ level of theory and corrected for basis-set superposition error (Boys & Bernardi, 1970, Simon *et al.*, 1996) (BSSE) and dispersion via the so-called Grimme correction (Grimme, 2004, 2006). The results for selected motifs, as shown in Figures S3.5, are gathered in Table S3.2.

Importantly, the above-mentioned calculations were first attempted using theory levels of lower complexity, which yielded comparable, although overall highly variable results. The Rh⋯Rh distance for isolated molecules, predicted by DFT calculations on different theory levels is shown in Table S3.3. The choice of CAM-B3LYP functional was made over others (*i.e.* PBE0 (Adamo & Barone, 1999, Ernzerhof & Scuseria, 1999), B3LYP (Becke, 1993, Lee *et al.*, 1988, Miehlich *et al.*, 1989)) due to its implementation of long-range interaction corrections (Yanai *et al.*, 2004). Atomic basis set of LANL2DZ was at first tried as the basis set for the entire molecule, although its precision turned out insufficient for proper analysis of higher excited singlet states (S_1, S_2, S_3), as the optimized geometries vastly overestimated positional changes occurring by the molecule ligands. Ultimately, a custom-made mixed basis set combining a more robust 6-31G** basis set (for light atoms) and LANL2DZ (for rhodium atoms), along with a Grimme correction for dispersion, was chosen. The experimentally obtained information about actual molecule properties (both spectroscopic and photocrystallographic) was in this case necessary in order to make a decision to implement a more complex theory level, further underlining the importance of a coordinated theory-experiment approach presented here.

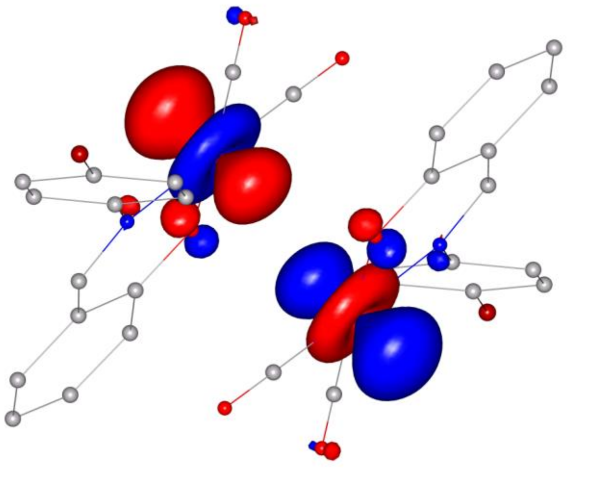

HOMO–2

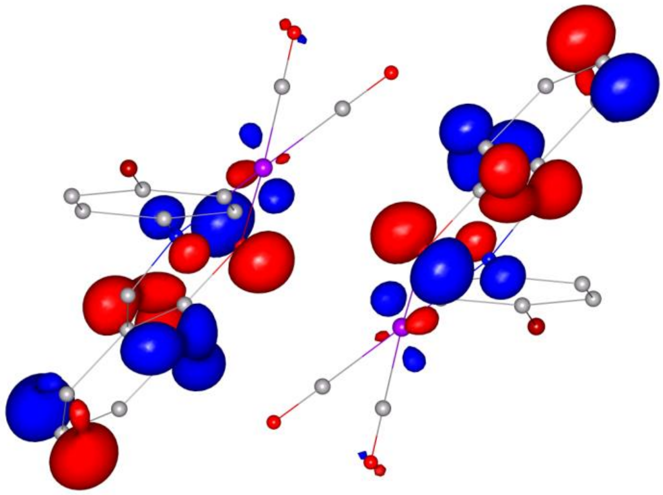

HOMO–1

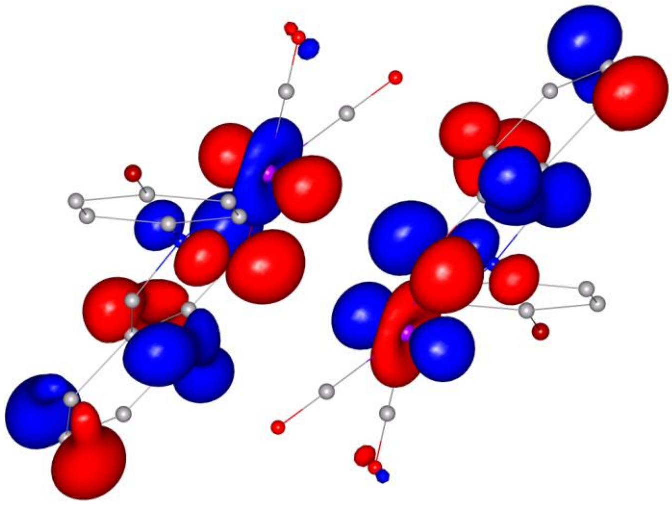

HOMO

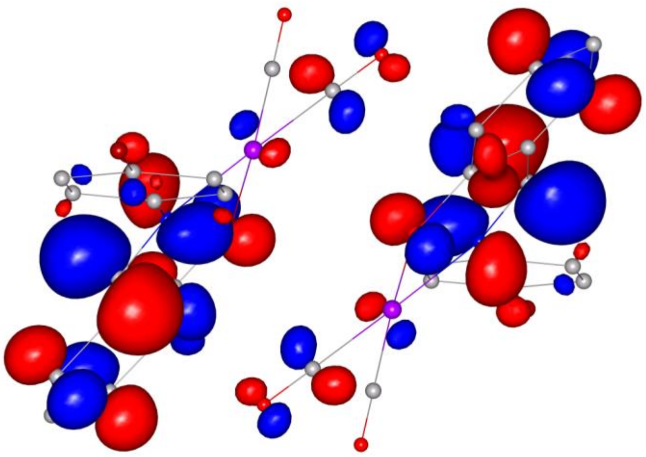

LUMO

Figure S3.1. Main molecular orbitals contributing to the $S_0 \rightarrow S_1$, $S_0 \rightarrow T_1$ and $S_0 \rightarrow S_3$ electronic transitions calculated at the DFT(CAM-B3LYP)/6-31G**-LANL2DZ theory level, showing MLCT character. All orbitals are drawn at the ±0.04 $e \cdot a_0^{-3}$ contour level, blue surfaces – positive, red – negative.

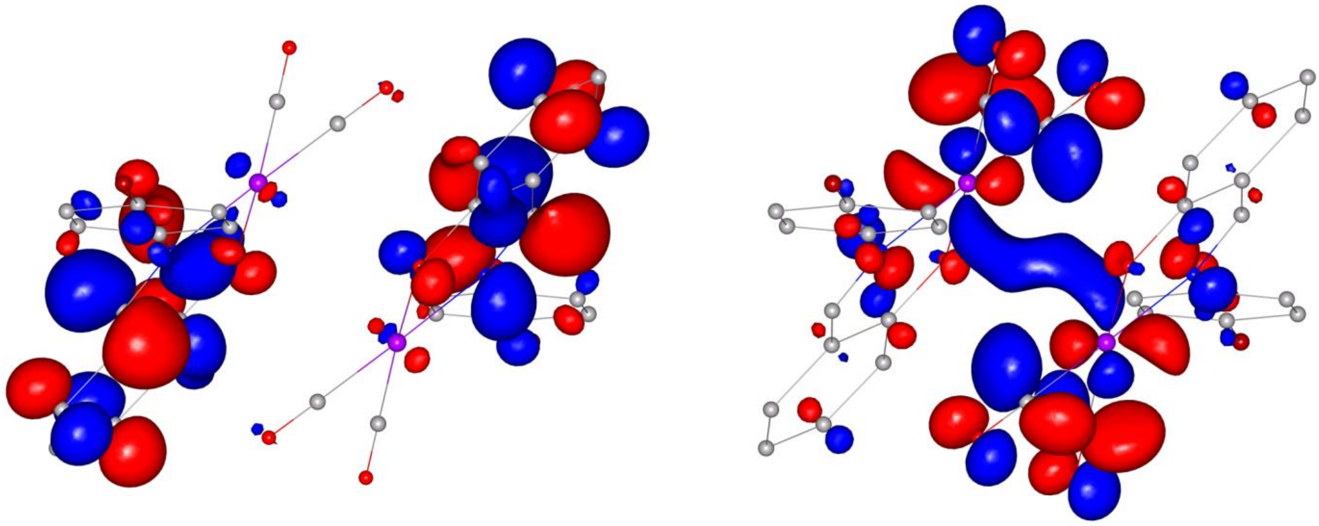

LUMO+1 LUMO+2

Figure S3.1 (continued). Main molecular orbitals contributing to the $S_0 \rightarrow S_1$, $S_0 \rightarrow T_1$ and $S_0 \rightarrow S_3$ electronic transitions calculated at the DFT(CAM-B3LYP)/6-31G**-LANL2DZ theory level, showing MLCT character. All orbitals are drawn at the ±0.04 $e{\cdot}a_0^{-3}$ contour level, blue surfaces – positive, red – negative.

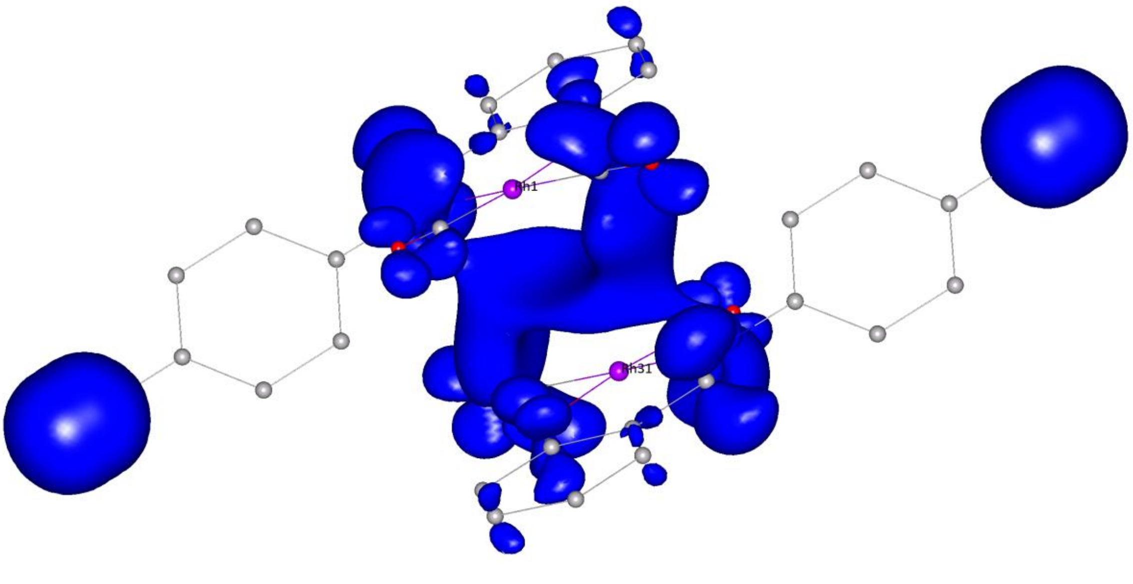

Figure S3.2. (S_1–S_0) transition-density map calculated at the DFT(CAM-B3LYP)/6-31G**-LANL2DZ level of theory, drawn at +0.0005 $e{\cdot}a_0^{-3}$ contour level, showing an influx of electron density in the general region of the Rh···Rh space in the **Rh-4-Br** excimer.

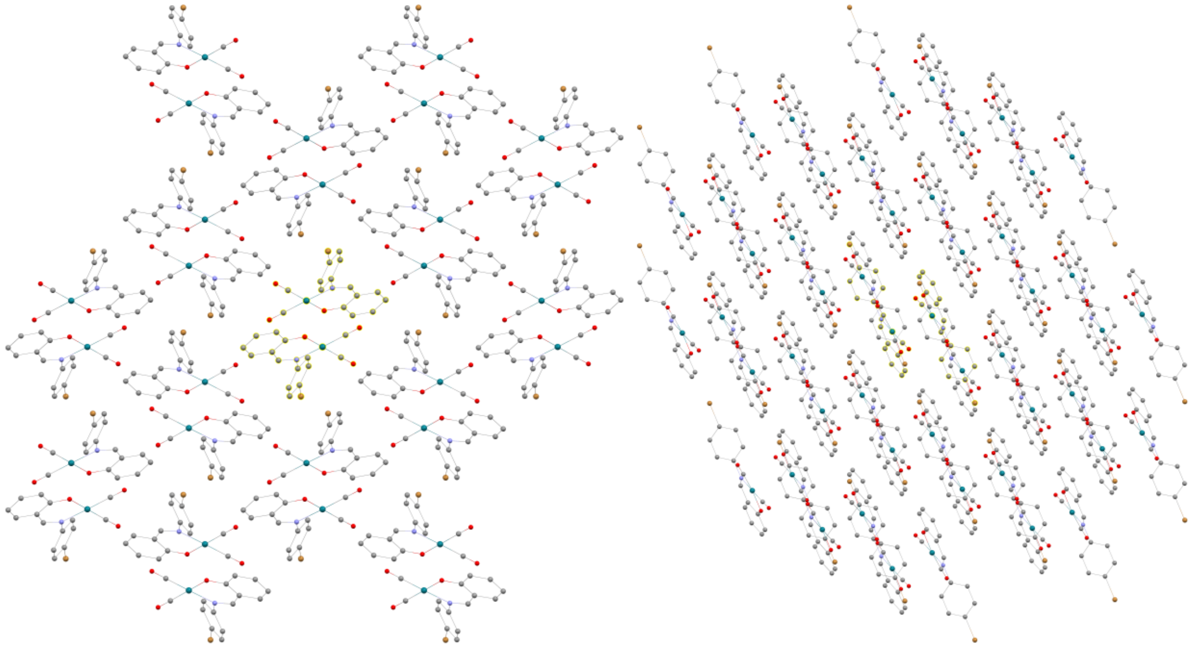

View along X axis View along Y axis

Figure S3.3. Molecular cluster used for QM/MM calculations. The highlighted dimer in the center was calculated at high level of theory (DFT(CAM-B3LYP)/6-31G**-LANL2DZ), while the molecular shell was treated at low level (UFF) level of theory. The cluster was generated so that all atoms of the central molecule have their complete atomic environment within a radius of 15 Å.

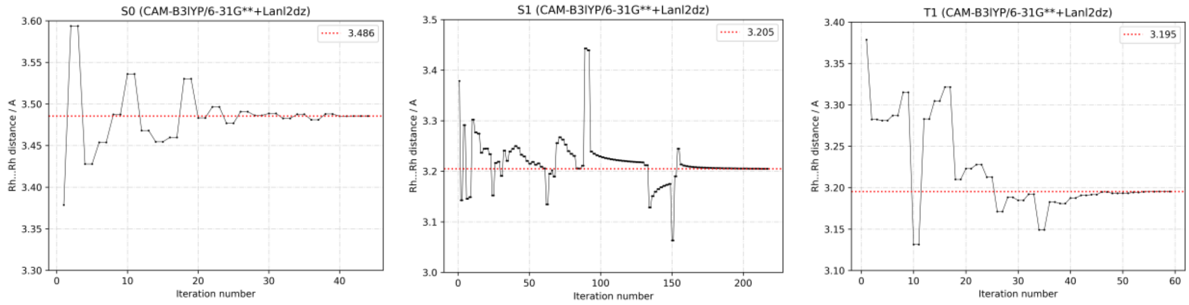

S_0 (ground state) S_1 state T_1 state

Figure S3.4. QM/MM at DFT(CAM-B3LYP)/6-31G**-LANL2DZ/UFF theory level geometry optimization process overview with regard to the Rh···Rh distance at each optimization step. The red dotted line represents the value for the final converged model.

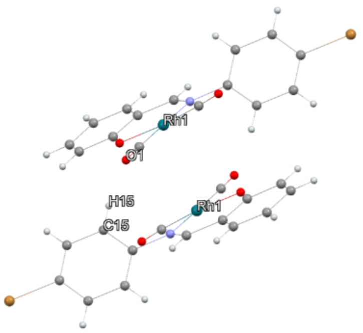

D1

(Rh···Rh interaction supported by weak C15–H15···O1 interaction)

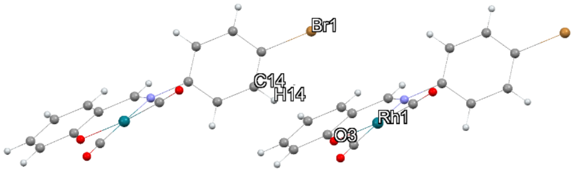

D2

(Rh···Br interaction supported by weak C14–H14···O3 interaction)

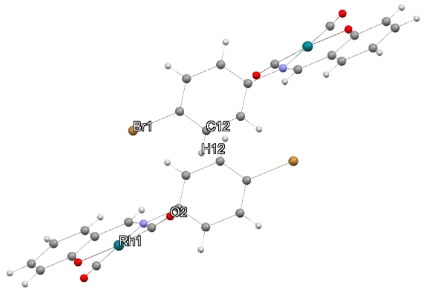

D3

(Rh···Br interaction supported by weak C12–H12···O2 interaction)

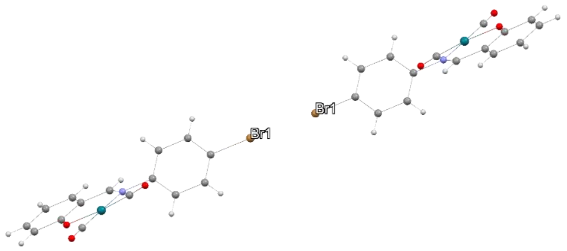

D4

(weak Br···Br interaction)

Figure S3.5. Dimeric motifs (**D1–D4**) present in the **Rh-4-Br** crystal structure. Main interactions are commented.

Table S3.1. Considered electronic transitions along with molecular orbitals with major contributions (MOs).

Transition	*Energy, E* / eV	*Wavelength, λ* / nm	*Oscillator strength, f*	*MOs with major contributions*	*MO contribution*
$S_0 \rightarrow S_1$	3.4597	358.37	0.0959	HOMO−2 → LUMO+2	26.01%
				HOMO−2 → LUMO	22.04%
				HOMO → LUMO	15.81%
				HOMO → LUMO+2	13.49%
$S_0 \rightarrow S_2$	3.5567	348.59	0.0 [a]	HOMO → LUMO+1	37.46%
				HOMO−1 → LUMO	29.30%
$S_0 \rightarrow S_3$	3.5976	344.63	0.2070	HOMO → LUMO	41.44%
				HOMO−1 → LUMO+1	20.43%
				HOMO−2 → LUMO+4	10.80%
$S_0 \rightarrow T_1$	2.6047	475.99	0.0 [a]	HOMO → LUMO	47.21%
				HOMO−1 → LUMO+1	37.23%

[a] Forbidden transition.

Table S3.2. Dimer interaction energies (E_{int}) evaluated for selected dimeric motifs from the **Rh-4-Br** GS (ground state) and ES (excited state) crystal structures.

Motif	E_{int} / kJ mol^{-1}	*Interaction*	*Distance, d* / Å
**D1**-GS	−74.18	Rh···Rh	3.379(4)
**D1**-ES	−79.45		3.19(1)
**D2**-GS	−26.28	Rh···Br	4.348(5)
**D2**-ES	−26.15		4.46(1)
**D3**-GS	−40.88	Rh···Br	4.206(5)
**D3**-ES	−41.63		4.32(1)
**D4**-GS	−3.77	Br···Br	3.521(4)
**D4**-ES	−3.77		3.521(4)

Table S3.3. Calculated Rh···Rh distance in an isolated dimer.

Level of theory	*Rh···Rh distance,* $d_{\text{Rh}\cdots\text{Rh}}$ / Å		
	S_0 state	T_1 state	S_1 state
PBE0/LANL2DZ [a]	3.649	2.903	2.803
PBE0/LANL2DZ	3.375	2.785	2.764
B3LYP/def2SVP	3.408	2.833	2.797
B3LYP/6-31G**-LANL2DZ	3.344	2.924	2.825
CAM-B3LYP/def2SVP	3.618	2.806	2.919
CAM-B3LYP/6-31G**-LANL2DZ	3.495	2.816	2.971
CAM-B3LYP/6-31G**-SDD [b]	3.616	2.814	2.939

[a] Without the G3DBJ Grimme dispersion correction. [b] SDD = Stuttgart/Dresden basis set (Fuentealba *et al.*, 1982) (only for Rh atoms, as in the case of LANL2DZ basis set).

Table S3.4. Calculated Hirschfeld charges (selected atoms are shown) for the QM/MM-optimized S_0 and S_1 electronic states of **Rh-4-Br** using DFT(CAM-B3LYP)/6-31G**-LANL2DZ level of theory.

Atom	*Charge,* Q_{H} / e		*Charge change,* ΔQ_{H} / e
	S_0 state	S_1 state	
Rh1	+0.056	+0.074	+0.018
O1	−0.095	−0.080	+0.016
O2	−0.107	−0.110	−0.003
O3	−0.249	−0.270	−0.022
N1	−0.087	−0.096	−0.009

4. Solid-state spectroscopy

Firstly, the solid-state UV-Vis absorption spectrum was collected for a powdered **Rh-4-Br** sample mixed with KBr and formed into a thin pellet. The respective measurement was performed using a Shimadzu UV-2600i UV-Vis spectrometer, result of which is shown in Figure S4.1.

Solid-state emission spectra of **Rh-4-Br** in the solid state were measured for a single crystal sample at multiple temperatures, including room temperature, 250 K, 200 K, 150 K, and 100 K, using a custom-built setup established at the Department of Chemistry, University of Warsaw. The setup consists of a tunable Ekspla NT230 Nd:YAG DPSS laser, an Olympus IX73 inverted microscope, a Cryo Industries of America (CIA) microscope cryocooler stage, and a Princeton Instruments SP-2150 spectrograph equipped with a PI-MAX4 iCCD detector. The single-crystal of **Rh-4-Br** was transferred onto a very thin (*ca.* 0.1 mm) 1 cm diameter circular quartz plate with a minimal amount of the Type-F Olympus immersion oil, placed inside the microscope stage, and gradually cooled to the desired temperature. Luminescence decay measurements were conducted with 100 ps intervals, extending up to 20 ns, or until no signal beyond background noise was detected. Crystals were excited with λ_{ex} = 390 nm laser pulses. The resulting decay curves (Figure S4.3) were subsequently fitted with an exponential decay function to estimate excited-state lifetimes. For all temperatures, higher-exponential fitting did not yield additional distinct lifetimes, or did not converge at all. The stationary emission spectra for each temperature are illustrated in Figure S4.2. The maximum wavelength of emission (λ_{em}^{max}) has been assessed by averaging the value of the maximum wavelength for the first ⅙th of the frames collected. However, due to noticeably irregular shape of the emission curve around the top, as well as the non-uniform morphological nature of measured crystals, this maximum approximation method could be imprecise by about 5 nm to either side.

Additionally, in order to account for possible thermally activated delayed fluorescence (TADF), the gating time-width of the ICCD camera was set to a value of 1 ns, and a coarse scan was made starting from the –5 ns time delay (5 ns before laser pulse arrives) and ending on +1 ms time delay with 1 ns interval between frames. Within this scan only a clean decaying signal was registered, with no signs of delayed luminescence.

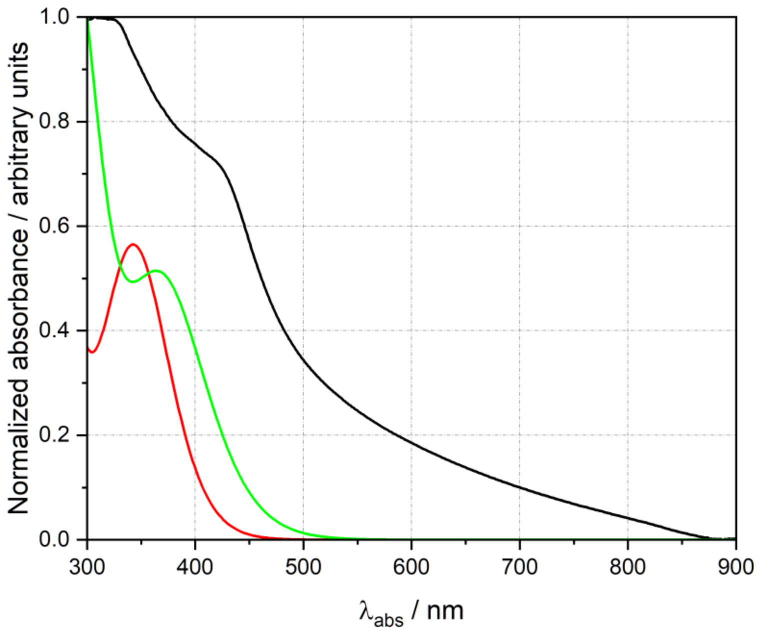

Figure S4.1. Normalized UV-Vis spectrum of the **Rh-4-Br** powder-diluted in KBr (black line), theoretical spectrum calculated at the DFT(CAM-B3LYP)/6-31G**-LANL2DZ (red), DFT(PBE0)/LANL2DZ (green) levels of theory.

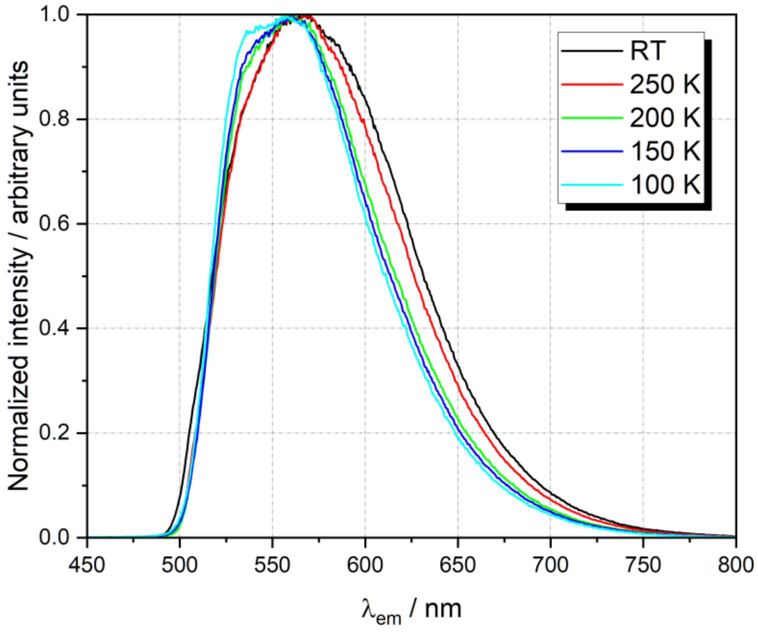

Figure S4.2. Normalized solid-state stationary emission spectra collected for the **Rh-4-Br** single crystal at selected temperatures.

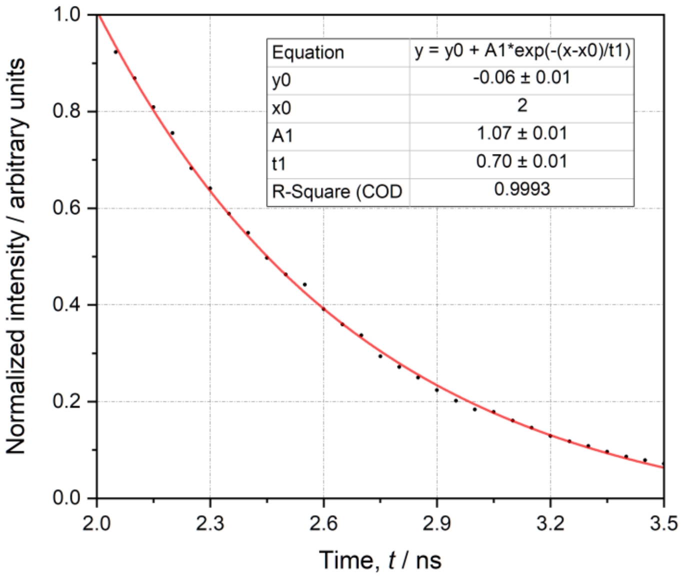

T = 293 K (λ_{em}^{max} = 566 nm)

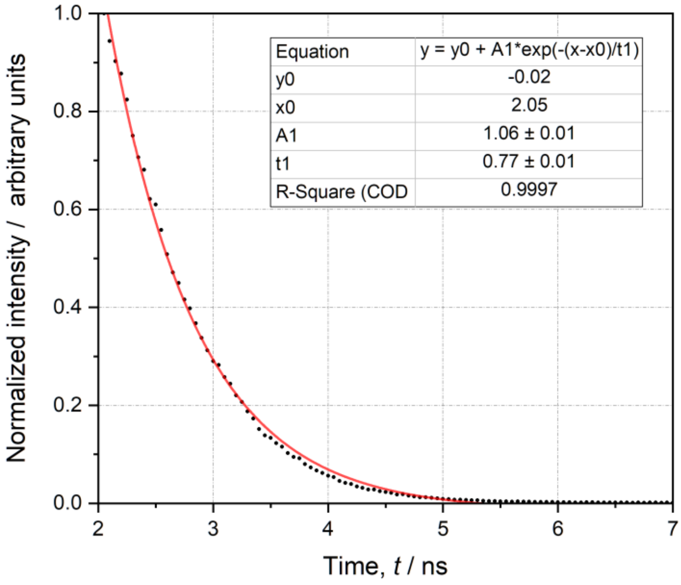

T = 250 K (λ_{em}^{max} = 561 nm)

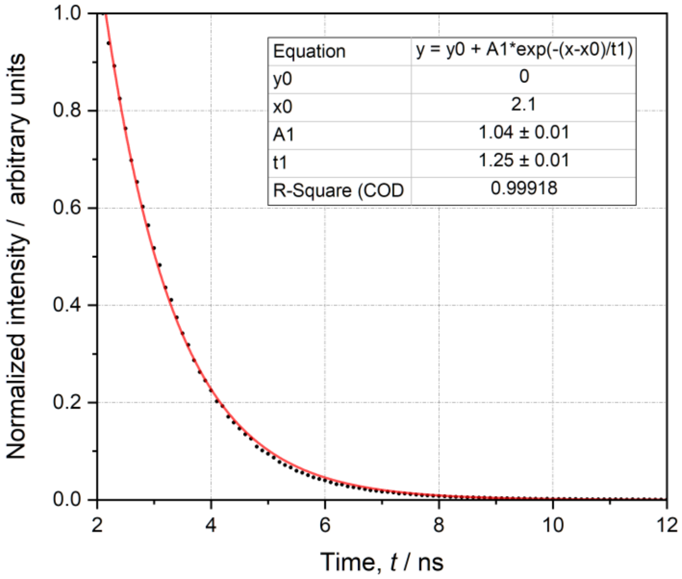

T = 200 K (λ_{em}^{max} = 558 nm)

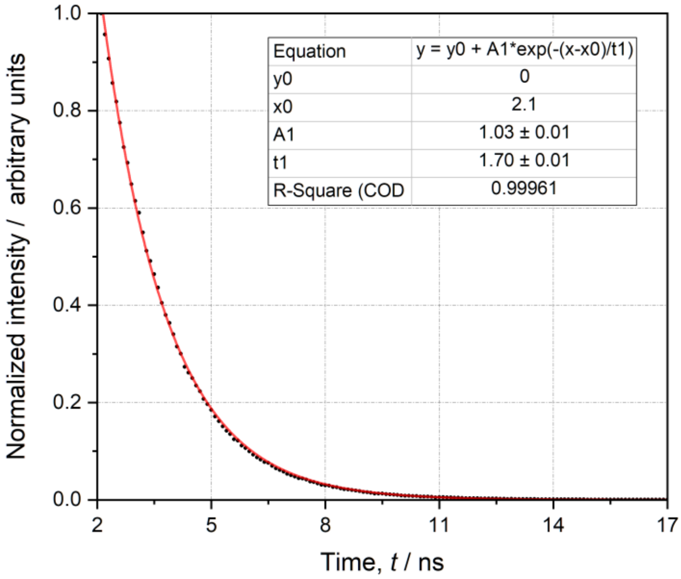

T = 150 K (λ_{em}^{max} = 558 nm)

Figure 4.3. Luminescence decay curves measured at various temperatures after λ_{ex} = 390 nm laser light excitation. In all cases the central maximum of emission is given.

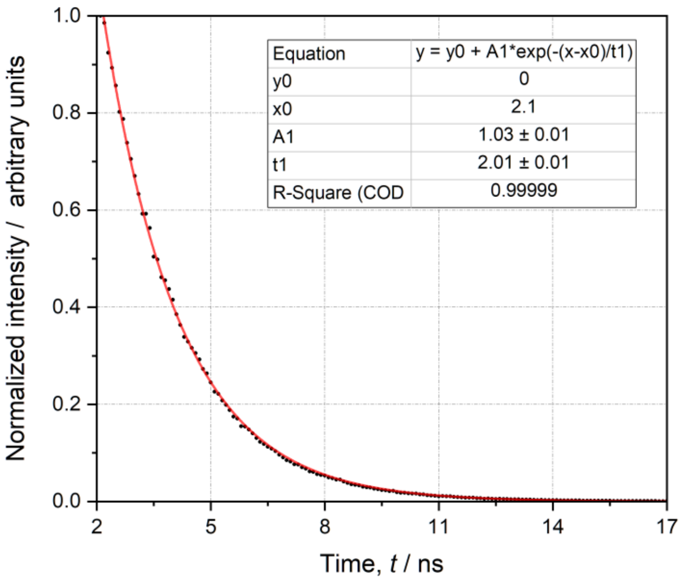

T = 100 K (λ_{em}^{max} = 557 nm)

Figure 4.3 (continued). Luminescence decay curves measured at various temperatures after λ_{ex} = 390 nm laser light excitation. In all cases the central maximum of emission is given.

5. Time-resolved X-ray Laue diffraction

5.1. Data collection. The time-resolved Laue diffraction experiments involved laser-pump/X-ray-probe measurements conducted at the 14-ID-B BioCARS beamline, at the Advanced Photon Source (APS) in Chicago, Illinois, USA (Graber *et al.*, 2011). During data collection, single crystal samples were mounted on glass fibers and kept at a constant temperature of 100 K with the use of nitrogen cryostream device. The X-ray radiation had a polychromatic wavelength range of approximately 0.8 Å to 1.0 Å (15 keV undulator setting; maximal intensity of the polychromatic 'pink' beam), with each X-ray pulse lasting about 80 ps. The channel-cut scan plot is presented in Figure S5.1. For photoexcitation of crystals, an on-site Ti:Sapphire picosecond laser was used (λ = 390 nm, beam size (fwhm): 76 μm$_h$ × 68 μm$_v$, pulse duration: 38 ps), with the laser power density ranging between 1.0 and 13.2 μJ per pulse. Crystals were of a rod-like shape and cut to about 20−50 μm linear size. The penetration depth, estimated by the solid-state UV-Vis measurement at 390 nm wavelength, was around 30 μm, thus well matched the crystal sizes. In order to maximize the sample response while keeping the laser-induced deterioration to minimum, optimal laser power setting was found for each sample by conducting preliminary short scans and laser-power-to-signal correlation plot analysis

(Coppens *et al.*, 2017). The delay between the laser pump and the X-ray probe was set to 100 ps. To account for long-range fluctuations in the X-ray beam's position and intensity, 5 pairs of successive light-ON and light-OFF frames were recorded for each goniometer angular setting before proceeding to the next position. The details on the collection strategy for all data sets are summarized in Table S5.1. Directly before the pump-probe experiment, each sample was also measured in the absence of laser to facilitate further orientation-matrix determination.

5.2. Data processing. Bragg peaks on each frame collected during the experiments were integrated and filtered with the use of the *LAUEPROC* software toolkit (Szarejko *et al.*, 2020, Kamiński *et al.*, 2020), which utilizes seed-skewness signal-searching algorithm, suited specifically for time-resolved studies of small molecules (signal level parameter: s = 3.0, trust level parameter: t = 0.2). The resulting intensities were then indexed for each frame independently, utilizing the refined orientation matrices obtained by using the locally-modified *LAUEUTIL* software suite (Kalinowski *et al.*, 2012, Kalinowski *et al.*, 2011). Next, for each dataset, the light-ON and light-OFF intensity ratios of indexed reflections were derived by means of statistical analysis of the recurring (ON/OFF) pairs, utilizing the RATIO method (Coppens & Fournier, 2015a, Coppens *et al.*, 2009): $R_{\mathrm{o}} = I_{\mathrm{ON}}/I_{\mathrm{OFF}}$ (subscript "o" stands for "observed"). The datasets were then rescaled according to literature-reported procedure (Fournier *et al.*, 2016, Coppens & Fournier, 2015b) and merged using the *SORTAV* program (Blessing, 1987, 1997, Blessing & Langs, 1987). The relevant statistics of merged datasets are collected in Tables S5.2 and S5.4. The completeness of the collected and used Laue data compared with full data completeness of the monochromatic experiment is visualized in Figure S5.8.

5.3. Photodifference maps. In order to plot the photodifference maps (Fournier & Coppens, 2014), the merged and scaled dataset containing 1464 unique reflections (51.2% data completeness) underwent Fourier difference synthesis against $F_{\mathrm{o}}^{\mathrm{ON}}$and $F_{\mathrm{o}}^{\mathrm{OFF}}$ structure factors, where the $F_{\mathrm{o}}^{\mathrm{OFF}}$ structure factors were obtained from the reference in-house monochromatic X-ray diffraction measurement (thus abbreviation 'mOFF'), and $F_{\mathrm{o}}^{\mathrm{ON}}$ were estimated from the intensity ratios obtained in the Laue experiment with the formula $F_{\mathrm{o}}^{\mathrm{ON}}(\mathbf{h}) = \sqrt{R_{\mathrm{o}}(\mathbf{h})} \cdot F_{\mathrm{o}}^{\mathrm{mOFF}}(\mathbf{h})$. The resulting photodifference map (Figure S5.2, S5.3, S5.4) is defined as follows:

$$\Delta\varrho_{\mathrm{pdiff}}(\mathbf{r}) = \frac{1}{V}\sum_{\mathbf{h}} \left(\sqrt{R_{\mathrm{o}}(\mathbf{h})} \cdot |F_{\mathrm{o}}^{\mathrm{mOFF}}(\mathbf{h})| - |F_{\mathrm{o}}^{\mathrm{mOFF}}(\mathbf{h})|\right) e^{\mathrm{i}\phi_{\mathrm{c}}^{\mathrm{mOFF}}(\mathbf{h})} e^{2\pi\mathrm{i}\mathbf{hr}},$$

where summation goes over all collected reflections, and superscript "c" denotes "calculated" (*i.e.* the phases are taken from the prior monochromatic-experiment model).

5.4. Refinement. The response-ratio ($\eta = R - 1$) refinement was conducted with the *LASER* program (Vorontsov *et al.*, 2010), where the central rhodium atom position, state population and the temperature scale factor were obtained for the excited-state measured in the Laue experiment. To improve the signal-to-noise ratio, the experimentally obtained reflections ratios were filtered by the criteria of $|1 - R_\mathrm{o}|/\sigma(R_\mathrm{o})$ ≥ 0.5 and $\sigma(R_\mathrm{o})$ ≤ 0.25. As the internal program procedure failed to converge when estimating excited-state population (due to the high correlations), it had to be estimated semi-manually, by tracking the change of the program-defined R_R-factor goodness metric for the refinement against different preset population values (Figure S5.5). The R_R-factor is defined as follows (Coppens *et al.*, 2010):

$$R_R = \frac{\Sigma|R_\mathrm{o} - R_\mathrm{c}|}{\Sigma R_\mathrm{o}},$$

where sums go over all measured reflections. In this way, the excited-state population was estimated to be around 1%, while the temperature scale factor ($U_{ij}^{\mathrm{ON}} = k_\mathrm{B} \cdot U_{ij}^{\mathrm{OFF}}$) was estimated at k_B = 1.075 (Table S5.3, Figure S5.7). Due to relatively low excited-state population, for the purpose of rhodium atom position refinement, the rest of the molecule was assumed to be rigid with the same geometry as determined based on the in-house X-ray diffraction measurement. The rhodium-atom shift is illustrated in Figure S5.6. The Rh⋯Rh distance in the refined excited-state model amounted to 3.190(10) Å, which is 0.189 Å shorter when compared to the ground-state case (3.379(4) Å).

In addition, another time-resolved Laue experiment was conducted with the pump-probe delay set to 1 ns, in order to monitor the excited-state population time-evolution. Three of the collected datasets have been processed in the manner identical to 100 ps delay dataset, and the resulting photodifference map is shown in Figure S5.4. The negative signal around the heavy atoms, similar to the one observed in the 100 ps dataset, is still present, however, there is no statistically significant electron density influx in the region between Rh atoms. This suggests that the excited-state population, which was not very high to begin with, decreases below the point of detection in the timespan of the first nanosecond after photoexcitation with 390 nm laser light.

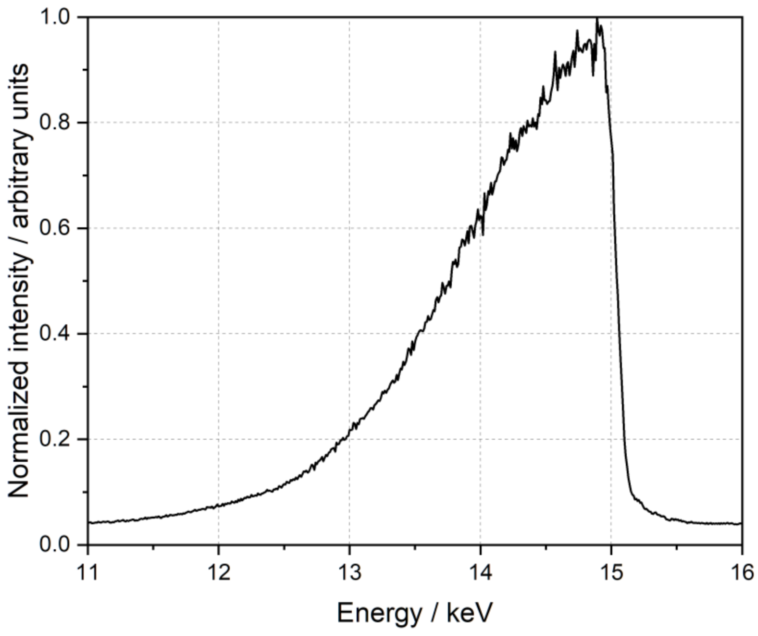

Figure S5.1. Channel-cut scan plot showing the spectral width and distribution of 'pink' (*i.e.* narrow-band) X-ray beam used during the current Laue experiment.

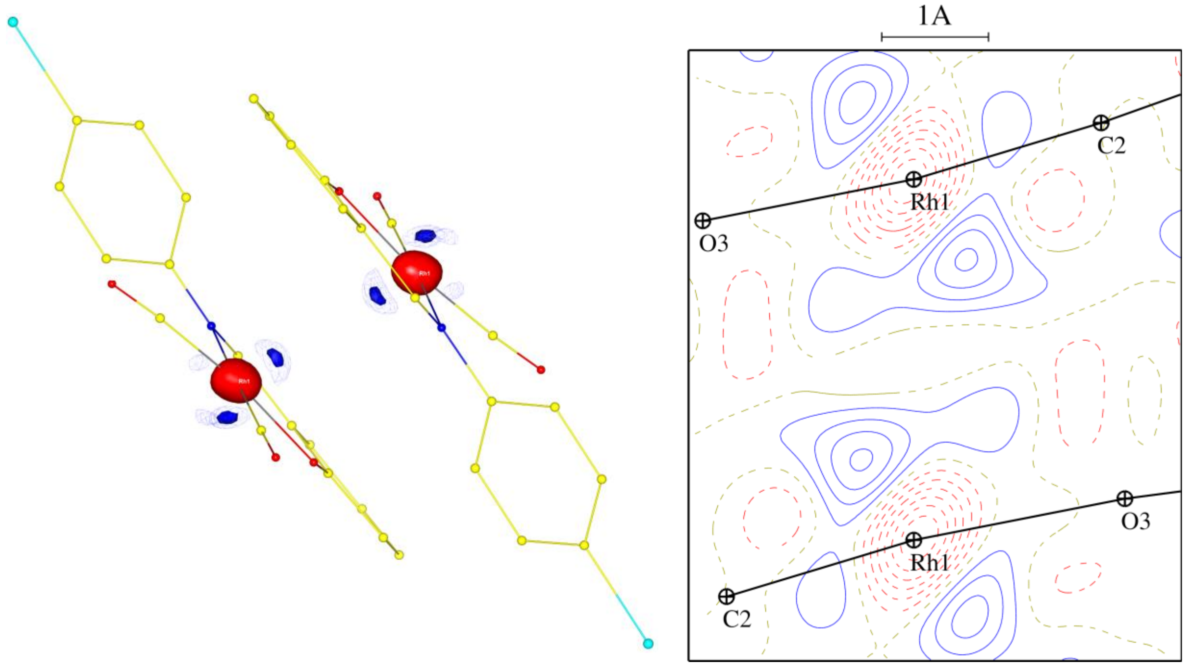

(*a*) (*b*)

Figure S5.2. Photodifference map obtained by plotting the electron-density changes on the **Rh-4-Br** dimer ground state geometry: (*a*) 3D visualization, where solid blue and red isosurfaces represent influx or reduction of electron density of at least ±0.5 e·Å^{-3} and blue transparent mesh represents influx of electron density of at least ±0.4 e·Å^{-3}. (*b*) Contour map, where contours represent an increment of electron density of ±0.1 e·Å^{-3}.

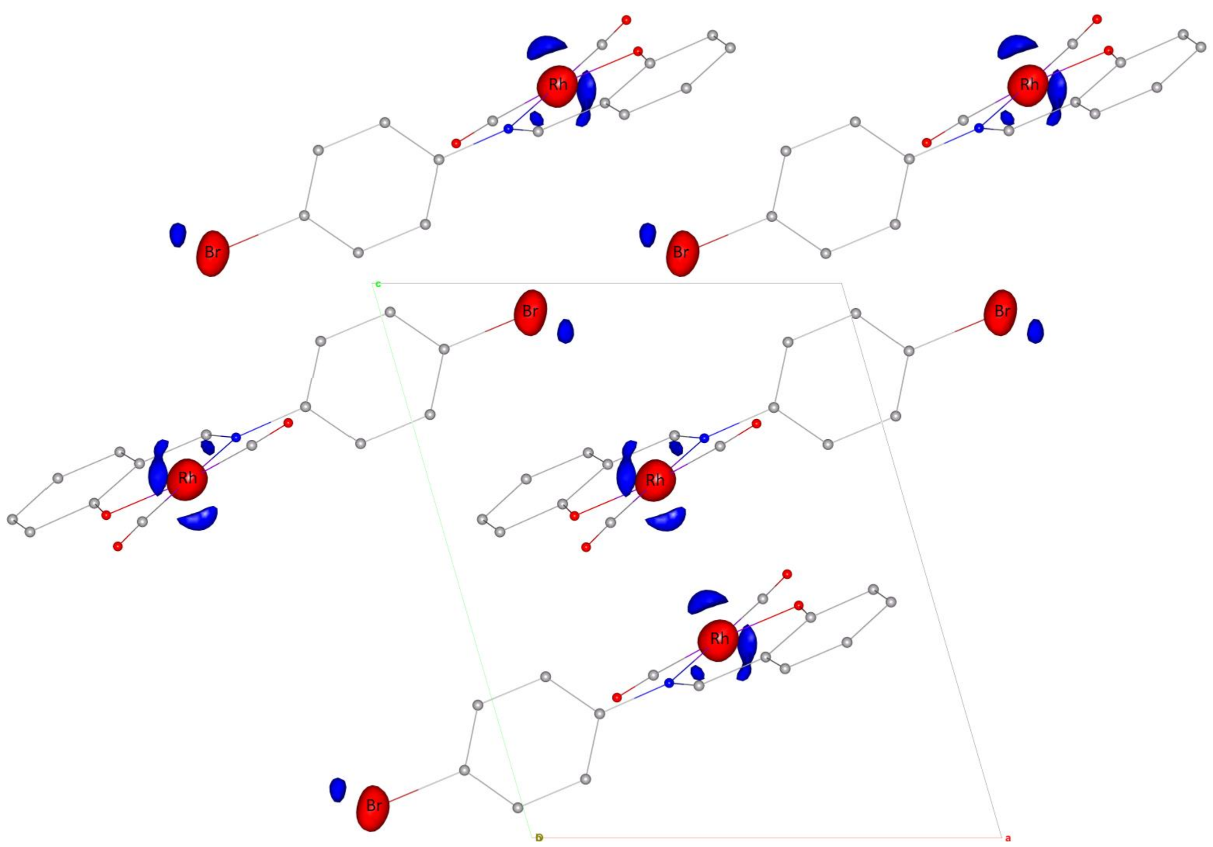

Figure S5.3. Photodifference map obtained by plotting the electron-density changes on the **Rh-4-Br** ground state geometry measured 100 ps after excitation, showcasing a possible Rh···Br distance shortening (not examined in this study). Blue and red isosurfaces represent influx or reduction of electron density of at least 0.4 e·Å^{-3}.

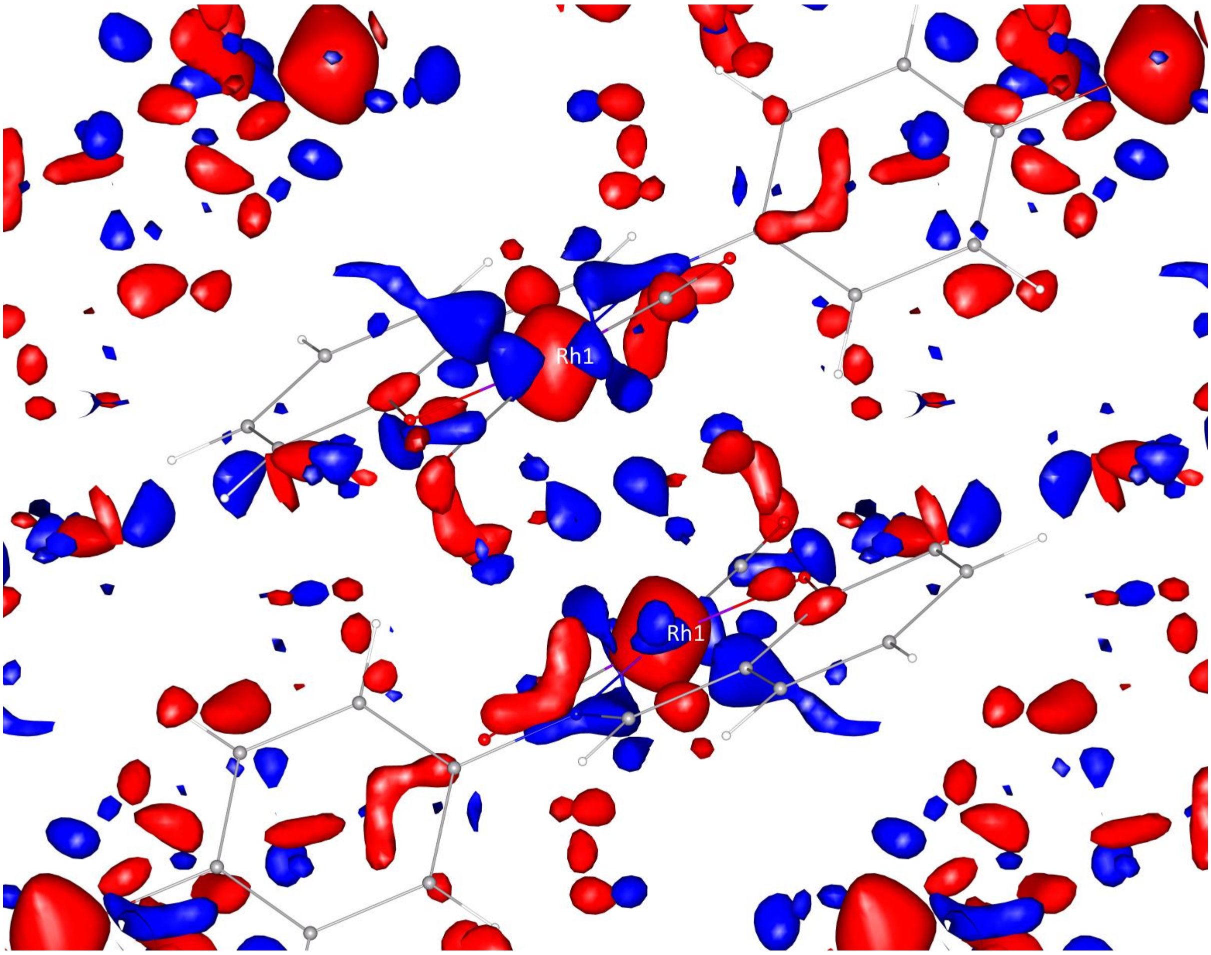

Figure S5.4. Photodifference map obtained by plotting the electron-density changes on the **Rh-4-Br** ground state geometry measured 1 ns after excitation, containing mostly noise. Blue and red isosurfaces represent influx or reduction of electron density of at least 0.35 e·Å^{-3}.

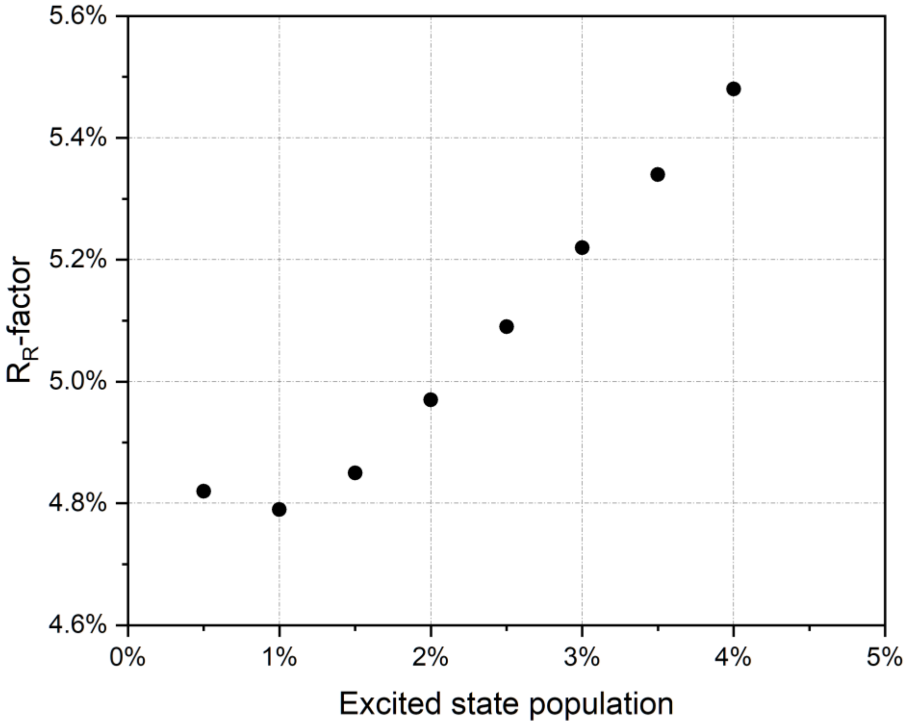

Figure S5.5. R_R-factor parameter calculated for each refinement model with different set excited-state population. The lowest value (R_R = 4.79%) was obtained the excited-state population of 1%.

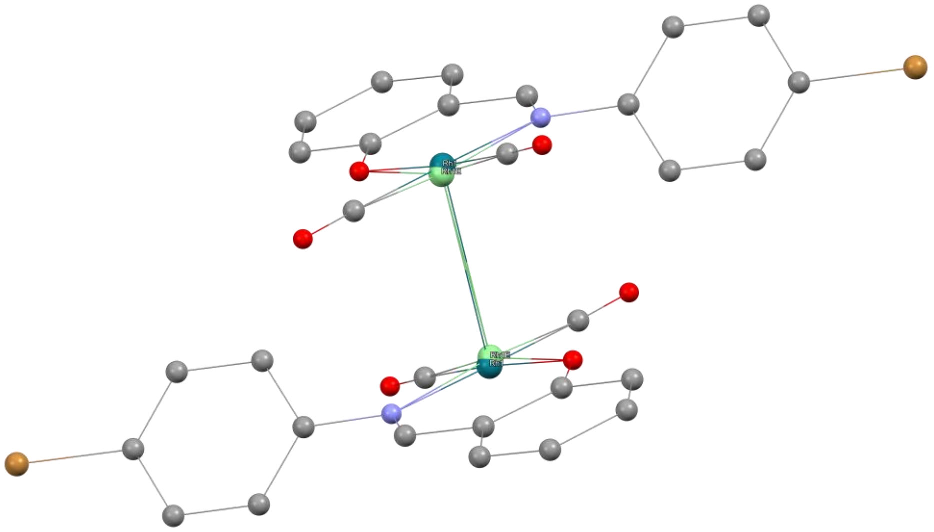

Figure S5.6. Refined **Rh-4-Br** excited-state Rh atoms' positions (light green) plotted on the **Rh-4-Br** ground-state geometry. The Rh···Rh distance for the ground state is 3.379(4) Å, while for the excited state it is 3.190(10) Å, showing a shortening of approximately 0.189(14) Å.

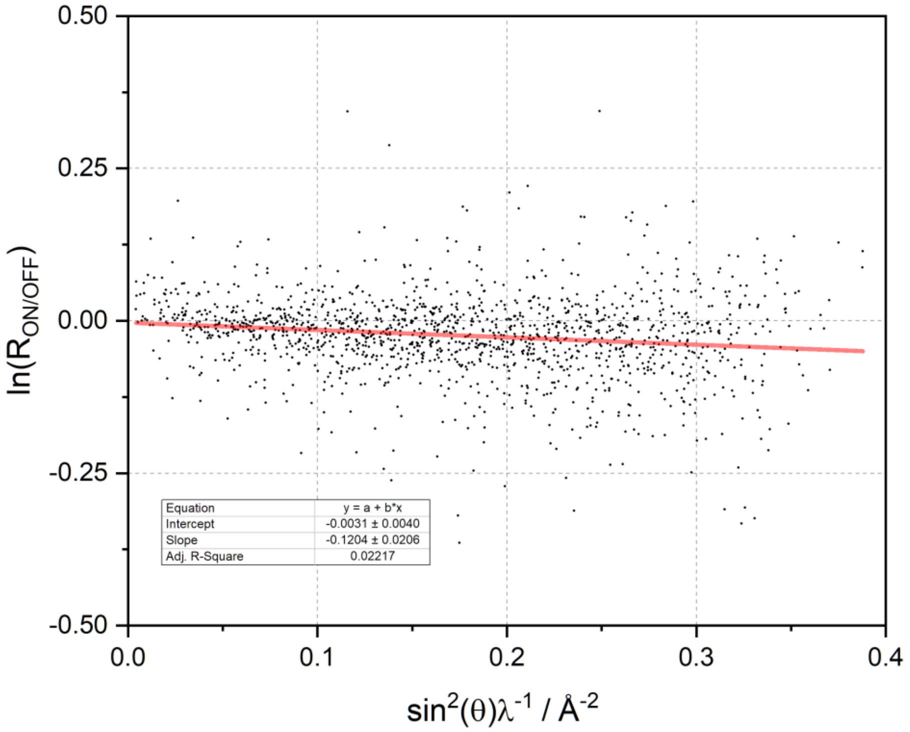

Figure S5.7. Photo-Wilson plot generated based on the collected TR Laue data. The temperature rise upon excitation can be estimated to *ca.* 4 K.

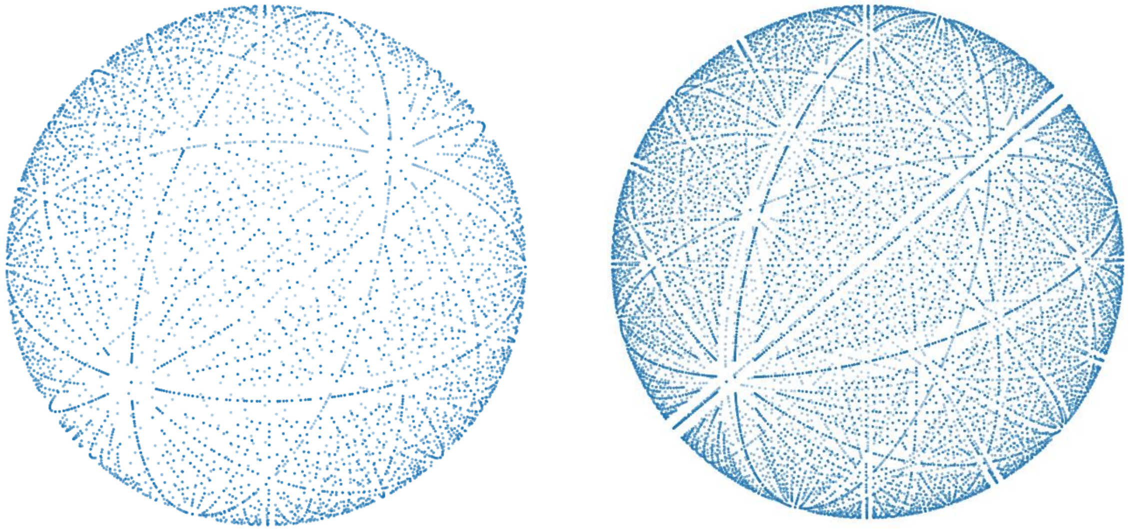

(a) (b)

Figure S5.8. Visualization of all collected reflections in reciprocal space projected on a unit sphere. The orientation of the sphere is not aligned whereas the symmetry equivalent reflections have been generated to account for the $2/m$ Laue class symmetry: (a) merged dataset collected during the Laue experiment (51.2% data completeness); (b) dataset collected during the in-house monochromatic experiment.

Table S5.1. Data collection strategy for the Laue experiment.

Data set	*Crystal size* / μm^3	*Strategy*	N_{frm}	φ_{tot} / °	φ_{inc} / °	N_{pp}	P_1 / mJ·mm^{-2}	P_2 / μJ·pulse^{-1}
Set 1	50 × 50	5×(ON,OFF)	910	0.0–180.0	2.0	12	3.6	13.2
Set 2	108 × 44	5×(ON,OFF)	910	0.0–180.0	2.0	12	1.0	4.4
Set 3	55 × 26	5×(ON,OFF)	910	0.0–180.0	2.0	12	1.0	3.7
Set 4	31 × 21	5×(ON,OFF)	910	0.0–180.0	2.0	5	0.25	1.0

Table legend: N_{frm} – total number of collected frames, φ_{tot} – total angular coverage φ_{inc} – inter-frame increment, N_{pp} – number of laser-pump X-ray-probe cycles per single frame, P_1 & P_2 – laser power.

Table S5.2. Collected dataset statistics and maximal resolution.

Data set	*No. of unique reflections*	*Data completeness*	$(\sin\theta/\lambda)_{\mathrm{max}}$ / Å^{-1}
Set 1	475	19.6%	0.59
Set 2	1062	37.1%	0.62
Set 3	1239	46.2%	0.61
Set 4	594	23.3%	0.60
Merged	1464	51.2%	0.62

Table S5.3. R_R-factor calculated for each refinement model with differing set excited-state population, P.

ES population, P	R_R (%)
0.5	4.82
1.0	4.79
1.5	4.85
2.0	4.97
2.5	5.09
3.0	5.22
3.5	5.34
4.0	5.48

Table S5.4. Distribution of measured and missing reflections in equal-volume resolution shells ($s = \sin\theta / \lambda$, $d = (2s)^{-1}$).

s_{max} / Å^{-1}	d_{min} / Å	*No. of measured reflections*	*No. of missing reflections*	*Completeness* (%)
0.229	2.179	129	9	93.5
0.289	1.730	131	16	89.1
0.331	1.511	125	18	87.4
0.364	1.373	123	17	87.9
0.392	1.275	117	30	79.6
0.417	1.199	112	39	74.2
0.439	1.139	99	46	68.3
0.459	1.090	83	50	62.4
0.477	1.048	96	61	61.1
0.494	1.012	81	44	64.8
0.510	0.980	70	84	45.5
0.525	0.952	72	63	53.3
0.539	0.927	56	90	38.4
0.553	0.904	59	79	42.8
0.566	0.884	41	91	31.1
0.578	0.865	30	133	18.4
0.590	0.848	22	137	13.8
0.601	0.832	11	84	11.6
0.612	0.817	4	184	2.1
0.623	0.803	3	101	2.9

6. References

Adamo, C. & Barone, V. (1999). *J. Chem. Phys.* **110**, 6158-6169.

Allen, F. H. (2002). *Acta Cryst. Sect. B* **58**, 380-388.

Allen, F. H. & Bruno, I. J. (2010). *Acta Cryst. Sect. B* **66**, 380-386.

Allen, F. H., Kennard, O., Watson, D. G., Brammer, L., Orpen, A. G. & Taylor, R. (1987). *J. Chem. Soc., Perkin Trans. 2*, S1-S19.

Becke, A. D. (1993). *J. Chem. Phys.* **98**, 5648-5652.

Blessing, R. H. (1987). *Cryst. Rev.* **1**, 3-58.

Blessing, R. H. (1997). *J. Appl. Cryst.* **30**, 421-426.

Blessing, R. H. & Langs, D. A. (1987). *J. Appl. Cryst.* **20**, 427-428.

Boys, S. F. & Bernardi, F. (1970). *Mol. Phys.* **19**, 553-566.

Bruker AXS (2003). *COSMO.* Version 1.48.

Bruker AXS (2004). *SAINT-PLUS (including XPREP).* Version 7.12.

Bruker AXS (2016). *SADABS.* Version 2016/2.

Conradie, J., Cameron, T. S., S. Aquino, M. A., Lamprecht, G. J. & Swarts, J. C. (2005). *Inorg. Chim. Acta* **358**, 2530-2542.

Conradie, M. M. & Conradie, J. (2013). *Journal of Molecular Structure* **1051**, 137-143.

Conradie, M. M., van Rooyen, P. H., Pretorius, C., Roodt, A. & Conradie, J. (2017). *J. Mol. Struct.* **1144**, 280-289.

Coppens, P. & Fournier, B. (2015a). *J. Synchrotron Rad.* **22**, 280-287.

Coppens, P. & Fournier, B. (2015b). *Struct. Dyn.* **2**, 064101.

Coppens, P., Kamiński, R. & Schmøkel, M. S. (2010). *Acta Cryst. Sect. A* **66**, 626-628.

Coppens, P., Makal, A., Fournier, B., Jarzembska, K. N., Kamiński, R., Basuroy, K. & Trzop, E. (2017). *Acta Cryst. Sect. B* **73**, 23-26.

Coppens, P., Pitak, M., Gembicky, M., Messerschmidt, M., Scheins, S., Benedict, J. B., Adachi, S.-I., Sato, T., Nozawa, S., Ichiyanagi, K., Chollet, M. & Koshihara, S.-Y. (2009). *J. Synchrotron Rad.* **16**, 226-230.

Dolomanov, O. V., Bourhis, L. J., Gildea, R. J., Howard, J. A. K. & Puschmann, H. (2009). *J. Appl. Cryst.* **42**, 339-341.

Ernzerhof, M. & Scuseria, G. E. (1999). *J. Chem. Phys.* **110**, 5029-5036.

Fournier, B. & Coppens, P. (2014). *Acta Cryst. Sect. A* **70**, 291-299.

Fournier, B., Sokolow, J. & Coppens, P. (2016). *Acta Cryst. Sect. A* **72**, 250-260.

Frisch, M. J., Trucks, G. W., Schlegel, H. B., Scuseria, G. E., Robb, M. A., Cheeseman, J. R., Scalmani, G., Barone, V., Petersson, G. A., Nakatsuji, H., Li, X., Caricato, M., Marenich, A. V., Bloino, J., Janesko, B. G., Gomperts, R., Mennucci, B., Hratchian, H. P., Ortiz, J. V., Izmaylov, A. F., Sonnenberg, J. L., Williams, Ding, F., Lipparini, F., Egidi, F., Goings, J., Peng, B., Petrone, A.,

Henderson, T., Ranasinghe, D., Zakrzewski, V. G., Gao, J., Rega, N., Zheng, G., Liang, W., Hada, M., Ehara, M., Toyota, K., Fukuda, R., Hasegawa, J., Ishida, M., Nakajima, T., Honda, Y., Kitao, O., Nakai, H., Vreven, T., Throssell, K., Montgomery Jr., J. A., Peralta, J. E., Ogliaro, F., Bearpark, M. J., Heyd, J. J., Brothers, E. N., Kudin, K. N., Staroverov, V. N., Keith, T. A., Kobayashi, R., Normand, J., Raghavachari, K., Rendell, A. P., Burant, J. C., Iyengar, S. S., Tomasi, J., Cossi, M., Millam, J. M., Klene, M., Adamo, C., Cammi, R., Ochterski, J. W., Martin, R. L., Morokuma, K., Farkas, O., Foresman, J. B. & Fox, D. J. (2016). *GAUSSIAN 16.*

Fuentealba, P., Preuss, H., Stoll, H. & Von Szentpály, L. (1982). *Chem. Phys. Lett.* **89**, 418-422.

Graber, T., Anderson, S., Brewer, H., Chen, Y.-S., Cho, H., Dashdorj, N., Henning, R. W., Kosheleva, I., Macha, G., Meron, M., Pahl, R., Ren, Z., Ruan, S., Schotte, F., Šrajer, V., Viccaro, P. J., Westferro, F., Anfinrud, P. & Moffat, K. (2011). *J. Synchrotron Rad.* **18**, 658-670.

Grimme, S. (2004). *J. Comput. Chem.* **25**, 1463-1473.

Grimme, S. (2006). *J. Comput. Chem.* **27**, 1787-1799.

Groom, C. R., Bruno, I. J., Lightfoot, M. P. & Ward, S. C. (2016). *Acta Cryst. Sect. B* **72**, 171-179.

Hirshfeld, F. L. (1977). *Theor. Chim. Acta* **44**, 129-138.

Hopmann, K. H., Stuurman, N. F., Muller, A. & Conradie, J. (2010). *Organometallics* **29**, 2446-2458.

Huq, F. & Skapski, A. C. (1974). *J. Cryst. Mol. Struct.* **4**, 411-418.

Kalinowski, J. A., Fournier, B., Makal, A. & Coppens, P. (2012). *J. Synchrotron Rad.* **19**, 637-646.

Kalinowski, J. A., Makal, A. & Coppens, P. (2011). *J. Appl. Cryst.* **44**, 1182-1189.

Kamiński, R., Jarzembska, K. N. & Domagała, S. (2013). *J. Appl. Cryst.* **46**, 540-534.

Kamiński, R., Schmøkel, M. S. & Coppens, P. (2010). *J. Phys. Chem. Lett.* **1**, 2349-2353.

Kamiński, R., Szarejko, D., Pedersen, M. N., Hatcher, L. E., Łaski, P., Raithby, P. R., Wulff, M. & Jarzembska, K. N. (2020). *J. Appl. Cryst.* **53**, 1370-1375.

Lee, C., Yang, W. & Parr, R. G. (1988). *Phys. Rev. B* **37**, 785-789.

Leipoldt, J. G., Bok, L. D. C., Basson, S. S., van Vollenhoven, J. S. & Gerber, T. I. A. (1977). *Inorg. Chimi. Acta* **25**, L63-L64.

Miehlich, B., Savin, A., Stoll, H. & Preuss, H. (1989). *Chem. Phys. Lett.* **157**, 200-206.

Mochida, T., Torigoe, R., Koinuma, T., Asano, C., Satou, T., Koike, K. & Nikaido, T. (2006). *Eur. J. Inorg. Chem.* **2006**, 558-565.

Pretorius, C., Brink, A. & Roodt, A. (2014). *Z. Kristallogr. NCS* **229**, 371-372.

Pretorius, C. & Roodt, A. (2012). *Acta Cryst. Sect. E* **68**, m1451-m1452.

Rappé, A. K., Casewit, C. J., Colwell, K. S., III, W. A. G. & Skiff, W. M. (1992). *J. Am. Chem. Soc.* **114**, 10024-10035.

Schurig, V., Gaus, H., Scheer, P., Walz, L. & von Schnering, H. G. (1989). *Angew. Chem. Int. Ed.* **28**, 1019-1021.

Schurig, V., Pille, W., Peters, K. & Georg Von Schnering, H. (1985). *Mol. Cryst. Liq. Cryst.* **120**, 385-388.

Schurig, V., Pille, W. & Winter, W. (1983). *Angew. Chem. Int. Ed.* **22**, 327-328.

Sheldrick, G. M. (2008). *Acta Cryst. Sect. A* **64**, 112-122.

Sheldrick, G. M. (2015). *Acta Cryst. Sect. A* **71**, 3-8.

Simon, S., Duran, M. & Dannenberg, J. J. (1996). *J. Chem. Phys.* **105**, 11024-11031.

Storch, G., Spallek, M. J., Rominger, F. & Trapp, O. (2015). *Chem. Eur. J.* **21**, 8939-8945.

Stuurman, N. F., Meijboom, R. & Conradie, J. (2011). *Polyhedron* **30**, 660-665.

Szarejko, D., Kamiński, R., Łaski, P. & Jarzembska, K. N. (2020). *J. Synchrotron Rad.* **27**, 405-413.

Varshavsky, Y. S., Cherkasova, T. G., Galding, M. R., Korlyukov, A. A., Podkorytov, I. S., Gindin, V. A., Smirnov, S. N., Mazur, A. S. & Rubaylo, A. I. (2018). *J. Organomet. Chem.* **874**, 70-73.

Vorontsov, I., Pillet, S., Kamiński, R., Schmøkel, M. S. & Coppens, P. (2010). *J. Appl. Cryst.* **43**, 1129-1130.

Vreven, T., Byun, K. S., Komáromi, I., Dapprich, S., Montgomery, J. A., Morokuma, K. & Frisch, M. J. (2006). *J. Chem. Theory Comput.* **2**, 815-826.

Yanai, T., Tew, D. P. & Handy, N. C. (2004). *Chem. Phys. Lett.* **393**, 51-57.
